# Dramatic Increases in Obesity and Overweight Prevalence among Asian Subgroups in the United States, 1992–2011

**DOI:** 10.5402/2013/898691

**Published:** 2013-10-29

**Authors:** Gopal K. Singh, Sue C. Lin

**Affiliations:** ^1^US Department of Health and Human Services Health Resources and Services Administration, Maternal and Child Health Bureau, 5600 Fishers Lane, Room 18-41, Rockville, MD 20857, USA; ^2^US Department of Health and Human Services Health Resources and Services Administration, Bureau of Primary Health Care, 5600 Fishers Lane, Room 6A-55, Rockville, MD 20857, USA

## Abstract

We examined trends in adult obesity and overweight prevalence among major Asian/Pacific Islander (API) subgroups and the non-Hispanic whites from 1992 to 2011. Using 1992–2011 National Health Interview Surveys, obesity, overweight, and BMI differentials were analyzed by logistic, linear, and log-linear regression. Between 1992 and 2011, obesity prevalence doubled for the Chinese, the Asian Indians, the Japanese, and the Hawaiians/Pacific Islanders; and tripled for the Filipinos. Obesity prevalence among API adults tripled from 3.7% in 1992 to 13.3% in 2010, and overweight prevalence doubled from 23.2% to 43.1%. Immigrants in each API subgroup had lower prevalence than their US-born counterparts, with immigrants' obesity and overweight risks increasing with increasing duration of residence. During 2006–2011, obesity prevalence ranged from 3.3% for Chinese immigrants to 22.3% for the US-born Filipinos and 41.1% for the Native Hawaiians/Pacific Islanders. The Asian Indians, the Filipinos, and the Hawaiians/Pacific Islanders had, respectively, 3.1, 3.8, and 10.9 times higher odds of obesity than those of the Chinese adults. Compared with Chinese immigrants, the adjusted odds of obesity were 3.5–4.6 times higher for the US-born Chinese and the foreign-born Filipinos, 9 times higher for the US-born Filipinos and whites, 3.8–5.5 times higher for the US-born and foreign-born Asian Indians, and 21.9 times higher for the Native Hawaiians. Substantial ethnic heterogeneity and rising prevalence underscore the need for increased monitoring of obesity and obesity-related risk factors among API subgroups.

## 1. Introduction

Adult obesity rates have increased dramatically in the United States, with the prevalence having risen more than twofold during the past 35 years [1]. Marked increases in obesity prevalence have occurred among both males and females and across all racial/ethnic and socioeconomic groups [1–3]. Due to high prevalence, a rapidly increasing trend, large racial/ethnic and socioeconomic disparities, and an unfavorable international ranking, current obesity levels in both children and adults are seen as a major public health problem in the USA [1–5]. 

While trend and current data on obesity for US adults are routinely available for such major racial/ethnic groups as the whites, the blacks, and the Hispanics [1, 6], prevalence estimates for specific Asian/Pacific Islander (API) subgroups are less well analyzed, particularly temporal obesity patterns among them [2]. Only a few studies have examined obesity differentials among APIs at the national level [2, 7–10], and obesity trends, focusing on API subgroups, remain unexplored [2]. The National Health and Nutrition Examination Survey (NHANES), the primary source for measured height, weight, and obesity statistics in the USA, does not even identify the overall API group let alone report data for specific API groups [2, 11]. The statistics for APIs are often presented under the residual category of “all other races.”

The API population, which comprises nearly 6% of the total US population, increased fivefold between 1980 and 2011 [12–16]. Indeed, the major API groups such as the Asian Indians, the Chinese, the Filipinos, the Koreans, and the Vietnamese are among the fastest growing racial/ethnic groups in the USA [12–16]. Increases in immigration from Asia following changes in the Immigration Act in 1965 have accounted for most of the rise in the API population [2, 12, 14, 17, 18]. More than 28% of the 40.4 million immigrants living in the USA in 2011 came from Asia [13, 19]. While immigrants account for 13% of the total USA population, they account for two-thirds of the API population [13, 19]. 

Given the unprecedented population increase, analysis of obesity patterns among immigrants of various Asian/Pacific Islander ethnicities assumes a special importance [2, 9]. Monitoring health inequalities according to race/ethnicity, immigrant status, and socioeconomic status (SES) has long represented an important area of research and policy analysis in the USA [1, 2, 9, 20–24]. Despite marked improvements in overall health and life expectancy, health inequalities in the USA have not only remained substantial but have also increased over time [9, 21–23]. Inequalities in chronic disease risks such as obesity, smoking, physical inactivity, and poor diet have contributed greatly to the persistence and/or widening of the health gradients [1, 2, 21–23]. The purpose of this study was to examine trends in obesity and overweight prevalence and body mass index (BMI) among major API subgroups and to identify those subgroups who are at high risk of obesity and who have experienced substantial increases in their obesity rates. Specifically, we (1) estimate changes in obesity and overweight prevalence over time among major API subgroups using large, nationally representative samples of US adults and (2) examine the extent to which obesity and overweight prevalence and BMI vary among specific API subgroups and how their obesity risks compare with those for the non-Hispanic white population. Since immigration is a major characteristic of the API population, our analysis is also stratified by nativity status to highlight immigrant differences in obesity rates within each group and across Asian-American ethnicities.

## 2. Methods

Temporal individual-level data on obesity, overweight, BMI, and selected socioeconomic, demographic, and behavioral characteristics were derived from the 1992–2011 National Health Interview Surveys (NHIS) [25, 26]. Descriptive sociodemographic data for various racial/ethnic groups were also derived from the 2011 American Community Survey (ACS) [13]. The NHIS, which is conducted by the National Center for Health Statistics, uses a complex, multistage probability design and is representative of the civilian noninstitutionalized population of the USA [1, 2, 9, 25, 26]. The household response rate for an annual NHIS generally exceeds 85%. All data are based on self-reports, including height and weight information, and obtained via in-home personal interviews [1, 25, 26]. Substantive and methodological details of the NHIS are described elsewhere [1, 25, 26]. 

Annual trends in obesity and overweight prevalence and BMI were estimated for the overall API category, non-Hispanic whites, and for all US adults from 1992 to 2011. To analyze trends over time for specific API subgroups, we pooled 4 years of the NHIS data from 1992 to 1995 and 6 years of data from 2006 to 2011. Aggregating data for several years in this fashion ensured sufficient sample sizes for analyzing patterns for groups stratified by Asian ethnicity, immigrant status, and SES. 

Obesity and overweight differentials were analyzed for 242,523 API and non-Hispanic white adults during 1992–1995 and 151,013 API and non-Hispanic white adults aged ≥18 during 2006–2011 for whom information on BMI was available. Adult overweight was defined as a BMI ≥ 25 kg/m^2^ and obesity as a BMI ≥ 30 kg/m^2^ [1, 2, 6, 27]. Note that the overweight category includes obese individuals.

During 1992–1995, race/ethnicity was classified into 9 major categories: the non-Hispanic whites, the Chinese, the Asian Indians, the Filipinos, the Japanese, the Koreans, the Vietnamese, the Hawaiians/Pacific Islanders, and a residual category of all other Asian/Pacific Islanders. During 2006–2011, data were not available separately for the Japanese, the Koreans, and the Vietnamese and were included in the residual other API group. Three ethnic-minority groups: blacks, American Indians/Alaska Natives, and Hispanics were not considered. Immigrant status was defined on the basis of adults' place of birth [2, 9, 10, 20]. US-born individuals were those born in one of the 50 states or Washington, DC. Immigrants or foreign-born people refer to those born outside these territories [2, 9, 10, 20]. The joint variable of ethnic-immigrant status included 12 categories, with each racial/ethnic group divided into the US-born and foreign-born people. Note that, although the Hawaiians are native-born, a small percentage of the Hawaiian/Pacific Islanders are foreign-born or born outside the 50 states and Washington, DC. The Pacific Islander immigrants consist of people born in Samoa, Guam, and other Pacific Islands [16, 18, 19].

In addition to race/ethnicity and immigrant status, we considered the following sociodemographic factors that are known to influence obesity: age, gender, marital status, region of residence, educational attainment (0–8, 9–11, 12, 13–15, and ≥16 years of school completed), family income/poverty status, and physical activity (PA) [2, 3, 9, 10, 27]. These covariates were measured as shown in Tables 1–3. Annual family income was also measured as a categorical variable; the income strata for 1992–1995 were <$7,000, 7,000–14,999, 15,000–19,999, 20,000–24,999, 25,000–34,999, 35,000–49,999, and ≥$50,000 [2]. 

PA level was measured by the number of times/week of vigorous activities of at least 10 minutes that caused heavy sweating or large increases in breathing or heart rate. The variable was coded as <1, 1-2, 3-4, and ≥5 times/week of activity. PA was not available during 1992–1995 [2, 26]. Prevalence of regular PA was defined for adults who engaged in at least 3 sessions per week of vigorous leisure-time physical activity lasting at least 20 minutes in duration or at least 5 sessions per week of light or moderate leisure-time physical activity lasting at least 30 minutes in duration [1]. 

Multivariate logistic regression was used to examine the association between the binary outcomes of obesity and overweight and selected socioeconomic and demographic factors. Least squares regression was used to model mean BMI. To account for the complex sample design of the NHIS, SUDAAN software was used to conduct all statistical analyses [28]. The two-sample *t*-test was used to test the difference in prevalence between any two groups at one point in time or to test for changes in prevalence between two time points for a specific group [2]. Log-linear regression models were used to estimate annual rates of increase in obesity/overweight trends in APIs, non-Hispanic whites, and the total US population [21, 22]. Specifically, the logarithm of the obesity and overweight rates was modeled as a linear function of time (calendar year), which yielded annual exponential rates of increase in prevalence [2, 21, 22]. 

## 3. Results

### 3.1. Annual Trends in Obesity and Overweight Prevalence, 1992–2011

**Figure 1:**
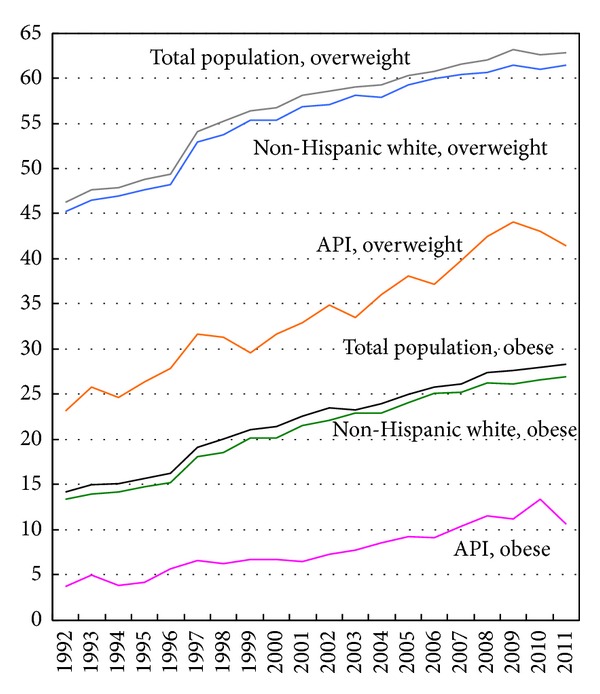
Trends in obesity and overweight prevalence (%) among the Asian/Pacific Islanders (API), the non-Hispanic whites, and the total US population aged 18+, 1992–2011.

The obesity prevalence for the total US adult population aged ≥18 doubled from 14.2% in 1992 to 28.3% in 2011. The overweight prevalence for all US adults increased from 46.3% in 1992 to 62.8% in 2011. Obesity prevalence among API adults tripled from 3.7% in 1992 to 13.3% in 2010, whereas overweight prevalence for API adults doubled from 23.2% to 43.1%. The obesity prevalence for the non-Hispanic white adults increased consistently from 13.3% in 1992 to 26.9% in 2011, whereas overweight prevalence for them increased from 45.2% to 61.4% during the same time period (Figure 1). During 1992–2011, the average annual rate of increase in obesity prevalence was 6.2% (95% CI = 5.4–7.0) for APIs, 4.0% (95% CI = 3.5–4.45) for the non-Hispanic whites, and 3.9% (95% CI = 3.4–4.3) for all US adults. The annual rates of increase in overweight prevalence were 3.3% (95% CI = 2.9–3.6) for APIs, 1.7% (95% CI = 1.4–1.9) for the non-Hispanic whites, and 1.7% (95% CI = 1.4–1.9) for all US adults. Thus, although obesity and overweight prevalence was substantially lower for APIs than for whites and the total population, the prevalence increased at a faster rate among APIs. The mean BMI for APIs increased from 22.9 in 1992 to 24.8 in 2011, while for the non-Hispanic whites it increased from 25.2 to 27.4 (data not shown). 

### 3.2. Socioeconomic and Demographic Profiles of API Groups in 2011

The API groups varied substantially in their socioeconomic characteristics (Table 1). Overall, APIs had higher SES levels than those of non-Hispanic whites. More than 18% of Hawaiians/Pacific Islanders were below the poverty line, compared with 13.7% of the Vietnamese, 12.2% of the Koreans, and 4.7% of the Filipinos. Socioeconomic attainment levels varied greatly within the API population. Approximately 15% of the Hawaiians/Pacific Islanders and 25.4% of the Vietnamese were college graduates, compared with 71.1% of the Asian Indians. Approximately 24% of the Hawaiians/Pacific Islanders and 30% of the Vietnamese were employed in professional and managerial occupations, compared with 67% of the Asian Indians. Median family income varied from a low of 54,485 for Hawaiians/Pacific Islanders to a high of 102,894 for the Asian Indians. More than two-thirds of the Asian Indian, Chinese, Filipino, Korean, and Vietnamese populations were foreign born. Nearly half of all the Vietnamese, the Chinese, and the Koreans were unable to speak English well, compared with ≤23% of the Asian Indians and Filipinos.

### 3.3. Racial/Ethnic Disparities in Obesity and Overweight Prevalence in 1992–1995 and 2006–2011

Table 1 shows significant increases in obesity and overweight prevalence between 1992–1995 and 2006–2011 for API subgroups, non-Hispanic whites, and the total population. Between 1992 and 2011, obesity prevalence doubled for the Chinese, the Asian Indians, the Japanese, and the Hawaiians/Pacific Islanders and more than tripled for the Filipinos. Overweight prevalence increased by 84% for the Filipinos and by 92% for the Vietnamese. 

In addition, considerable disparities in obesity and overweight prevalence among API subgroups and the non-Hispanic whites can be seen in Table 1. In 2006–2011, obesity prevalence varied from 4.4% for the Chinese to 10.4% for the Asian Indians, 16.5% for the Filipinos, and 39.6% for the Hawaiians/Pacific Islanders. The 2004–2006 data show an obesity prevalence of 8.7% for the Japanese and 5.3% for the Vietnamese. The 2006–2011 overweight prevalence was highest for the Hawaiians/Pacific Islanders (73.8%), followed by the non-Hispanic whites (60.8%), the Filipinos (53.2%), the Asian Indians (44.4%), the Japanese (34.6%), the Koreans (30.1%), and the Chinese (27.9%).

Regarding differences in obesity-related risk factors, the Chinese, the Asian Indians, the Filipinos, and the APIs as a whole were more likely to be physically inactive than the non-Hispanic whites. In terms of diet, substantial ethnic differences are noted, with the Asian Indians and the Chinese being significantly more likely to consume fruits and vegetables ≥1 times/day than non-Hispanic whites and the total population. Dietary information was not available for the Koreans, the Japanese, and the Vietnamese.

In 2006–2011, after adjusting for sociodemographic factors, the Asian Indians, the Filipino, the Hawaiians/Pacific Islanders, the non-Hispanic whites had, respectively 3.1, 3.8, 10.9, and 4.7 times higher odds of obesity than those of their Chinese counterparts. In 1992–1995, ethnic differentials in obesity were similar, with data for additional groups showing 67% lower odds of obesity among the Vietnamese and 89% higher odds of obesity among the Japanese compared to the Chinese. 

Risks of obesity and overweight increased with increasing duration in the United States (Tables 2 and 3). The obesity gradients by length of immigration were steeper in 2006–2011 than in 1992–1995. Compared with the US-born group, immigrants who had lived in the US for <1 year or ≥15 years had 66% or 26% lower odds of obesity in 2006–2011 and 49% or 26% lower odds of obesity in 1992–1995, respectively (Table 1). Immigrants who had lived in the US for <1 year or ≥15 years had 51% or 19% lower odds of overweight than the US-born group in 2006–2011 (Table 3).

### 3.4. Disparities in Obesity and Overweight Prevalence among API and White Immigrant Groups and US-Born Groups in 2006–2011

In 2006–2011, obesity prevalence ranged from 3.3% for Chinese immigrants to 22.3% for the US-born Filipinos, 26.4% for the US-born whites, and 41.1% for the Native Hawaiians/Pacific Islanders (Table 4). The overweight prevalence in 2006–2011 ranged from 25.2% for the Chinese immigrants to 57.6% for the US-born Filipinos, 61.1% for the US-born whites, and 72.4% for the Native Hawaiians/Pacific Islanders (Table 4). Mean BMI varied from a low of 23.1 for the Chinese immigrants to a high for 29.8 for the Native Hawaiians and 30.1 for the Pacific Islander immigrants (Table 4). 

Ethnic-immigrant differentials in obesity risks were greater than disparities shown by race/ethnicity alone (Table 4). Compared with Chinese immigrants, the adjusted odds of obesity were 3.5 to 4.6 times higher for the US-born Chinese and the foreign-born Filipinos, 8.8 to 9.5 times higher for the US-born Filipinos and the US-born whites, and 3.8 to 5.5 times higher for the US-born and the foreign-born Asian Indians. Compared with the Chinese immigrants, the odds of overweight were 2.4 times higher for the US-born Chinese, 4.7 times higher for the US-born Filipinos, 3.2 times higher for Filipino immigrants, 2.4 to 2.7 times higher for the US-born and foreign-born Asian Indians, and 3.6 to 4.5 times higher for white immigrants and natives (Table 3).

Socioeconomic gradients in obesity were steep, with education and income levels contributing independently to obesity and overweight risks during both 1992–1995 and 2006–2011 (Tables 2 and 3). During 2006–2011, those with less than a college education had at least 55% higher adjusted odds of obesity and at least 35% higher odds of overweight than those with a college degree. During 2006–2011, adults living below the poverty line had 32% higher adjusted odds of obesity than those with family incomes at ≥500% of the poverty threshold. 

Marked socioeconomic gradients in obesity and overweight prevalence were generally found for all racial/ethnic groups (Table 5). Obesity prevalence ranged from 4.3% for the Chinese in the highest SES category to 29.2% for the low-SES Filipinos, 32.5% for the low-SES whites, and 38.5% for the middle-SES Hawaiians/Pacific Islanders. Socioeconomic gradients in overweight prevalence and BMI were more consistent with the expected pattern. The high-SES Chinese had the lowest overweight prevalence of 24.4% and the low-SES Asian Indians, Filipinos, and Hawaiians/Pacific Islanders had the highest overweight prevalence of 62.9%, 63.6%, and 73.6%, respectively. Compared with the high-SES Chinese, the adjusted odds of obesity were, respectively, 8.8 and 14.7 times higher for the low-SES and middle-SES Hawaiians/Pacific Islanders, 12.4 times higher for the low-SES Filipinos, and 8.2 times higher for the low-SES whites. The low-SES Asian Indians, Filipinos, and Hawaiians/Pacific Islanders had 5.7, 8.6, and 13.3 times higher odds of overweight than high-SES Chinese, respectively (Table 5).

After adjusting for race/ethnicity and socioeconomic factors, physical inactivity was associated with 54% higher odds of obesity (OR = 1.54; 95% CI = 1.46–1.62) and 36% higher odds of overweight (OR = 1.36; 95% CI = 1.30–1.42) during 2006–2011 (data from the full models not shown).

## 4. Discussion

With obesity prevalence continuing to rise rapidly, it is important to analyze trends in obesity and related risk factors among the Asian/Pacific Islander Americans who constitute a significant part of the contemporary US work force and who account for more than 40% of all new immigrants arriving in the USA since 2008 [13, 19]. Obesity prevalence among all major API groups has increased sharply during the past two decades. These increases appear to be more marked than those observed in the other major racial/ethnic groups in the US such as the whites, blacks, American Indians/Alaska Natives, and Mexican Americans. The high obesity levels in the Hawaiians/Pacific Islanders and the alarming rise in obesity among the Filipinos and the Asian Indians are of particular concern. Approximately 40% of the Hawaiian/Pacific Islander adults are obese and 74% of them are either obese or overweight. The rates for the Hawaiian/Pacific Islanders are similar to those for the blacks and the American Indians/Alaska Natives who have an obesity prevalence of 40% and whose overweight prevalence exceeds 70% [1, 2, 26]. The Mexican-Americans and the Puerto Ricans are the two other US groups with relatively high obesity and overweight prevalence [2, 26]. 

Although recent trends indicate a slight decrease or leveling off in obesity/overweight prevalence among the APIs as a whole between 2009 and 2011, the absolute numbers of obese and overweight APIs keep rising. In 2011, there were an estimated 1.3 million obese and 5.0 million overweight API adults, compared to 0.2 million obese and 1.3 million overweight API adults in 1992. If the obesity trends of the past 20 years were to continue, our log-linear regression models forecast the obesity and overweight prevalence among API adults to increase to 22.0% and 60.1%, respectively, by 2020. For the total US adult population, the obesity and overweight prevalence in 2020 are projected to reach 43.2% and 76.6%, respectively (complete forecast data available from the authors).

As immigration to the US from Asia, especially from China, India, South Korea, and the Philippines, continues to increase in the future, the racial/ethnic and demographic composition of the US population is expected to become even more diverse, and routine monitoring of obesity and related risks among the APIs becomes increasingly important. Lower rates of obesity and overweight among the Chinese, the Asian Indians, the Filipinos, the Japanese, the Koreans, and the Vietnamese, compared to the whites and other ethnic groups, are consistent with their lower rates of morbidity and mortality from obesity-related diseases and chronic conditions [7, 9, 20]. Prevalences of heart disease, diabetes, and hypertension are higher in the Filipinos and Asian Indians than the Chinese and the Koreans [7]. The Native Hawaiians/Pacific Islanders, in fact, are more likely to suffer from heart disease, hypertension, stroke, and diabetes than any other racial/ethnic group in the United States [25, 29]. Life expectancy is higher and rates of mortality from cardiovascular diseases, colorectal cancer, and diabetes are considerably lower among the Asian-Americans as a whole and among the Chinese, the Japanese, and the Filipinos [9, 10, 20]. Rising prevalence of obesity among the APIs and whites is also consistent with the increasing trends in mortality and morbidity from diabetes seen among these groups and in the total US population [1, 9]. 

Substantial immigrant differentials in obesity risks among the Asian Americans shown here are consistent with those reported for nativity differentials for the total population and for various racial/ethnic groups [2, 8–10, 30]. Positive immigrant selectivity in health, education, and skills has been suggested as a possible explanation for lower obesity risks among immigrants [2, 9, 10]. Positive selection may also apply for Asian immigrants, who immigrate to the USA primarily under the skill criteria with high socioeconomic attainment levels [2, 9, 12, 19]. As shown here and previously, obesity and other health advantages for immigrants tend to diminish with increasing acculturation levels or length of residence in the USA [2, 8–10, 20, 30]. Acculturation effects, however, could vary by ethnicity. The lower obesity risks among the US-born Chinese and Asian Indians compared to US-born whites suggest that health advantages for certain Asian-American groups may persist into the second generation and beyond [2, 9, 20]. Although genetic factors may account for some of the racial/ethnic differences in obesity, the lower obesity rates of immigrants in most ethnic groups as compared with their US-born counterparts indicate the significance of social environments, acculturation, and lifestyle factors [2, 9, 20]. 

Declining physical activity levels, increasingly sedentary lifestyles, and increases in total energy intake have been cited as factors contributing to rising trends in adult obesity [1, 2, 31]. In analyses not shown here, we found that although increased leisure-time PA was associated with reduced obesity risks in both the APIs and whites, adjusting for PA levels had little impact on the magnitude of racial/ethnic differentials in obesity rates. Indeed, because the APIs had higher physical inactivity levels, adjusting for PA only widened racial/ethnic differentials in obesity. Use of public transport is associated with increased physical activity [31, 32]. As shown in Table 1, all API groups are more likely than whites to use public transport for commuting to work. The commuting patterns appear to be consistent with the racial/ethnic differentials in obesity reported here.

Differences in dietary factors may partly explain racial/ethnic and immigrant differentials in obesity shown here [2]. As shown in Table 1, the APIs have a higher intake of fruits and vegetables than the non-Hispanic whites and the total population. However, the Filipinos and the Hawaiians/Pacific Islanders are similar to the whites and are much less likely than the Chinese and the Asian Indians to consume fruits/vegetables daily. According to a recent study, immigrants in each racial/ethnic group had significantly lower total calorie and fat intake than those of their US-born counterparts [2]. Moreover, immigrants' likelihood of excess calorie and fat intake increased with longer duration of residence in the USA [2]. In this study, immigrants from the residual ethnic-immigrant category, who were primarily Asian immigrants, had the lowest total calorie and fat intake of all the ethnic-immigrant groups [2]. Although lower SES groups have higher consumption of lower-quality diets and energy-dense foods, data on these dietary outcomes are lacking for API subgroups [33, 34]. Future studies need to examine the impact of dietary variables in explaining obesity trends and differentials between racial/ethnic and immigrant groups shown here. 

This study has some limitations. Obesity and overweight prevalence estimates from NHIS are derived from self-reported height and weight data, which may underestimate the actual prevalence among various racial/ethnic, immigrant, and socioeconomic groups [2, 26]. During 2007–2010, for example, 27.8% and 63.4% of US adults were classified as obese and overweight, respectively, in the NHIS, whereas the corresponding NHANES prevalences were 34.9% and 68.7% [1, 11, 26]. Thus, the estimates presented may, at best, represent the lower bounds of the obesity and overweight burden in whites and the API subgroups. However, compared to the NHIS-based analyses, the NHANES with its much smaller sample size cannot permit detailed examinations of racial/ethnic, immigrant, and socioeconomic disparities in obesity such as those shown here. Second, dietary information in the NHIS is only available for selected years, such as 2005 and 2010, in which such data were collected as part of the Cancer Control Supplements [26]. NHIS data on immigration and acculturation are also limited. The NHIS does not collect information on legal status of immigrants as well as more direct measures of acculturation such as ethnic-cultural identity, language, social networks, and dietary preferences [2, 9, 10]. Finally, because of the small sample sizes we did not examine if obesity patterns and trends among API subgroups differed by gender; this needs to be examined in future studies. 

Social environments, such as socioeconomic status, as well as physical and built environments, are the underlying determinants of obesity [2, 23, 35–38]. They influence obesity risks through their effects on the proximate behavioral factors of diet, physical activity, and sedentary behaviors [2, 23, 35–38]. We have shown the significant impact of SES on obesity and overweight risks among both the APIs and the whites. Ethnic-minority and socially disadvantaged groups in the United States have lower access to neighborhood sidewalks, parks/playgrounds and green spaces, public transportation, and local grocery stores that carry healthy, affordable foods [23, 24, 35]. Health policy measures must address these broader social and physical environments as a part of a national strategy to prevent obesity and reduce health inequalities across social and racial/ethnic groups [23, 24, 35]. 

In conclusion, continued inequalities in obesity according to race/ethnicity, immigrant status, and SES will likely have substantial impacts on future obesity and chronic disease patterns in the United States [2]. The United States has the highest rates of obesity and overweight in the industrialized world [5, 27, 39]. Our analysis shows that even the highest-educated and wealthiest Asian Americans are not immune to the rising obesity trend. National efforts on obesity prevention and control must take into account considerable heterogeneity that exists among the APIs in terms of both obesity prevalence and the related social and behavioral determinants. The Asian Indians, the Filipinos, and the Hawaiians/Pacific Islanders have 2–10 times higher obesity and overweight prevalence than the Chinese. Except for Chinese immigrants, an average Asian American is likely to be overweight and the Native Hawaiian/Pacific Islander an obese adult. Indeed, the overall obesity and overweight prevalence for US adults and an overweight prevalence of 72% for the Native Hawaiians/Pacific Islanders and 76% for the Pacific Islander immigrants rank among the highest in the world [5, 27, 39]. Continued monitoring of disparities in obesity and overweight prevalence among the Asian/Pacific Islander Americans should, therefore, be an essential feature of the national strategy to track progress towards eliminating health inequalities among all the Americans [2, 40]. 

## Figures and Tables

**Table 1:** Selected sociodemographic and behavioral characteristics of Asian/Pacific Islander subgroups and the non-Hispanic whites: United States, 1992–2011.

Characteristic	Chinese	Asian Indian	Filipino	Korean	Vietnamese	Japanese	Hawaiian and Pacific Islander	Asian/ Pacific Islander (All)	Non-Hispanic white	Total US population
Obesity and related behaviors, 1992–2011										
Obesity prevalence (SE), 1992–1995	2.1 (0.33)	4.0 (0.61)	4.3 (0.66)	2.0 (0.53)	0.8 (0.25)	4.5 (0.70)	23.1 (3.15)	4.2 (0.49)	14.1 (0.11)	15.0 (0.11)
Obesity prevalence (SE), 1997–2000	3.6 (1.00)	6.2 (1.25)	8.4 (1.32)				24.2 (0.87)	6.3 (0.53)	19.3 (0.17)	20.4 (0.16)
Obesity prevalence (SE), 2006–2011	4.4 (0.59)	10.4 (1.09)	16.5 (1.10)	2.8 (0.97)	5.3 (1.54)	8.7 (2.07)	39.6 (3.52)	11.1 (0.47)	26.1 (0.22)	27.2 (0.19)
Increase in obesity prevalence (%), 1992–2011	105.7	161.4	242.0	42.9	606.7	92.5	71.3	164.4	85.3	81.5
Overweight prevalence (SE), 1992–1995	18.1 (0.81)	29.8 (1.58)	28.9 (1.36)	20.4 (1.43)	12.7 (1.00)	27.4 (2.17)	53.1 (4.33)	25.0 (0.88)	46.6 (0.16)	47.6 (0.16)
Overweight prevalence (SE), 1997–2000	22.5 (1.84)	32.9 (2.48)	39.7 (2.12)				61.0 (0.94)	30.7 (0.99)	54.3 (0.23)	55.6 (0.20)
Overweight prevalence (SE), 2006–2011	27.9 (1.32)	44.4 (1.50)	53.2 (1.44)	30.1	24.4	34.6	73.8 (2.96)	41.4 (0.82)	60.8 (0.23)	62.2 (0.20)
Increase in overweight prevalence (%), 1992–2011	54.5	49.1	84.2	47.33	92.13	26.51	39.0	65.5	30.6	30.5
Mean body mass index (BMI, SE), 1992–1995	22.4 (0.07)	23.5 (0.11)	23.6 (0.12)	22.5 (0.13)	21.7 (0.11)	23.14 (0.19)	26.8 (0.49)	23.1 (0.09)	25.3 (0.02)	25.5 (0.02)
Mean body mass index (BMI, SE), 2006–2011	23.4 (0.12)	24.8 (0.13)	26.0 (0.14)				29.9 (0.84)	24.8 (0.10)	27.3 (0.03)	27.5 (0.03)
Increase in BMI (%), 1992–2011	4.5	5.9	10.4				11.7	7.4	7.9	7.9
Physical activity prevalence (%), 2006–2011	62.3	62.6	60.1				56.5	61.6	58.1	60.4
Regular physical activity prevalence (%), 2006–2011	31.6	31.3	31.1				38.3	31.4	36.1	33.7
Fruit/vegetable intake <1 time/day (%), 2010	20.2	18.8	39.2				37.6	28.6	34.8	35.5
Sociodemographic characteristics, 2011										
Population size, single race	3,520,150	2,908,204	2,538,325	1,449,876	1,669,447	756,898	506,017	15,526,436	197,084,523	311,591,919
Population size, alone or in combination with other races	4,185,871	3,190,419	3,439,930	1,734,354	1,866,681	1,289,192	1,186,153	18,179,539	202,593,247	311,591,919
Median age (years)	38.1	32.5	39.5	37.0	36.6	48.6	29.3	36.1	42.3	37.3
Married-couple families (%)	57.8	73.1	58.3	53.6	60.3	46.1	51.5	60.6	51.4	48.3
Foreign-born (%)	69.7	71.2	66.5	73.5	67.0	38.8	22.1	65.2	3.9	13.0
Not speaking English “very well” (%)	46.6	21.9	23.2	44.9	53.3	22.6	13.8	36.1	1.6	8.7
High school graduate or higher (%)	81.2	90.8	92.3	92.0	69.4	94.8	85.1	85.1	91.1	85.9
Bachelor's degree or higher (%)	52.3	71.1	47.6	52.6	25.4	48.3	14.5	49.7	31.9	28.5
In labor force (%)	62.5	69.6	70.8	59.9	66.6	58.3	67.8	67.3	63.5	64.0
Unemployed (%)	4.1	5.3	5.7	4.3	6.0	3.0	9.3	5.0	5.3	6.5
Managerial and professional occupations (%)	54.4	66.5	42.4	46.1	29.7	53.6	24.0	47.5	40.1	36.0
Median family income ($)	77,209	102,894	85,222	61,452	58,520	90,550	54,485	80,101	69,715	61,455
Below poverty level (%)	11.3	4.8	4.7	12.2	13.7	4.5	18.3	10.5	11.0	11.7
Commuting to work—car, truck, or van—drove alone (%)	57.7	65.8	69.4	69.2	74.5	73.0	67.5	66.4	79.9	76.4
Commuting to work—public transportation (%)	16.3	11.7	9.0	9.0	3.2	7.7	5.6	10.4	3.0	5.0

Source: [13, 26].

Obesity and overweight data for the Koreans, the Vietnamese, and the Japanese are for 2004–2006 instead of 2006–2011. Fruit/vegetable intake data are from the 2010 NHIS Cancer Control Supplement.

Differences in obesity and overweight prevalence and BMI across racial/ethnic groups and time periods were statistically significant at *P* < 0.001.

**Table 2:** Adjusted odds and prevalence^1^ of obesity (BMI ≥ 30) among the Asian/Pacific Islanders and the non-Hispanic whites aged 18+ and by selected sociodemographic characteristics: United States, 1992–2011.

Covariates		1992–1995 (*N* = 242,523)				2006–2011 (*N* = 104,719)		
	OR	95% CI	%	SE	OR	95% CI	%	SE
Race/ethnicity								
Non-Hispanic white	4.77	3.40–6.69	13.9	0.1	4.66	3.53–6.15	25.5	0.2
Chinese	1.00	Reference	3.3	0.6	1.00	Reference	7.1	0.9
Asian Indian	2.15	1.38–3.35	6.8	1.1	3.14	2.20–4.48	19.0	2.0
Filipino	2.15	1.52–3.03	6.9	1.0	3.80	2.79–5.19	22.0	1.3
Korean	0.97	0.51–1.85	3.2	0.9				
Vietnamese	0.33	0.15–0.71	1.1	0.4				
Japanese	1.89	1.38–2.59	6.1	0.8				
Hawaiian/Pacific Islander	10.47	5.92–18.49	25.7	3.6	10.86	7.44–15.87	43.5	3.4
Other APIs^2^	3.79	2.53–5.68	11.4	1.7	1.72	1.25–2.35	11.5	0.8
Duration of residence in the USA (years)								
<1	0.51	0.31–0.85	7.7	1.8	0.34	0.17–0.67	10.8	3.3
1–5	0.72	0.55–0.93	10.4	1.2	0.37	0.26–0.54	11.6	1.9
5–9	0.45	0.37–0.55	6.9	0.6	0.49	0.37–0.64	14.6	1.6
10–14	0.57	0.46–0.71	8.5	0.8	0.47	0.36–0.60	14.1	1.5
15+	0.74	0.68–0.80	10.7	0.4	0.74	0.67–0.83	20.5	0.8
US-born	1.00	Reference	13.9	0.1	1.00	Reference	25.6	0.2
Age (years)								
18–24	1.00	Reference	6.5	0.2	1.00	Reference	13.4	0.5
25–34	1.94	1.81–2.08	11.8	0.2	1.90	1.72–2.09	22.4	0.5
35–44	2.67	2.50–2.86	15.4	0.2	2.64	2.38–2.92	28.5	0.5
45–54	3.51	3.29–3.75	19.3	0.2	2.77	2.51–3.06	29.4	0.4
55–64	3.22	3.01–3.46	18.0	0.3	3.02	2.70–3.36	31.2	0.5
65+	1.90	1.76–2.05	11.6	0.2	1.87	1.69–2.07	22.2	0.4
Gender								
Male	1.00	Reference	14.6	0.1	1.00	Reference	26.4	0.3
Female	0.86	0.84–0.88	12.8	0.1	0.86	0.83–0.90	23.7	0.3
Education (years of school completed)								
0–8	1.99	1.87–2.11	18.2	0.3	1.69	1.52–1.89	28.5	1.0
9–11	1.76	1.67–1.86	16.5	0.3	1.65	1.53–1.79	28.0	0.6
12	1.50	1.44–1.56	14.4	0.2	1.68	1.59–1.78	28.3	0.4
13–15	1.35	1.29–1.41	13.2	0.2	1.55	1.47–1.63	26.8	0.4
16+	1.00	Reference	10.2	0.2	1.00	Reference	19.3	0.3
Poverty status (ratio of family income to poverty threshold)^3^								
<100%	1.71	1.57–1.87	17.7	0.6	1.32	1.22–1.42	27.9	0.6
100–199%	1.62	1.52–1.72	16.9	0.3	1.35	1.26–1.44	28.4	0.5
200–299%	1.47	1.39–1.54	15.6	0.2	1.22	1.15–1.30	26.5	0.4
300–399%	1.32	1.26–1.38	14.3	0.2	1.22	1.14–1.31	26.6	0.6
400–499%	1.22	1.17–1.28	13.4	0.2	1.08	1.01–1.14	24.2	0.5
≥500%	1.00	Reference	11.3	0.2	1.00	Reference	22.9	0.4

OR: odds ratio; SE: standard error; CI: confidence interval.

^1^Adjusted prevalence was derived from fitted logistic models that included survey year, age, gender, race/ethnicity, length of immigration, marital status, region of residence, education, and poverty status (or family income and family size).

^2^Other Asian categories include the Koreans, the Vietnamese, the Japanese, the Cambodians, the Laotians, the Hmongs, the Thais, the Pakistanis, and other Asian groups.

^3^The income categories during 1992–1995 were <7000, 7000–14999, 15000–24999, 25000–34999, 35000–49999, and 50000+. Family income, instead of poverty status, was used for the 1992–1995 NHIS.

**Table 3:** Adjusted odds and prevalence^1^ of overweight (BMI ≥ 25) among the Asian/Pacific Islanders and the non-Hispanic whites aged 18+ and by selected sociodemographic characteristics: United States, 1992–2011.

Covariates	1992–1995 (*N* = 242,523)				2006–2011 (*N* = 104,719)			
	OR	95% CI	%	SE	OR	95% CI	%	SE
Race/ethnicity								
Non-Hispanic white	3.10	2.73–3.51	46.2	0.2	2.90	2.51–3.36	60.2	0.2
Chinese	1.00	Reference	23.3	1.1	1.00	Reference	36.1	1.6
Asian Indian	2.03	1.71–2.40	36.8	1.9	2.39	2.03–2.82	55.8	1.6
Filipino	2.00	1.70–2.35	36.6	1.6	2.83	2.39–3.36	59.7	1.3
Korean	1.27	1.06–1.52	27.5	1.6				
Vietnamese	0.66	0.53–0.82	17.0	1.4				
Japanese	1.71	1.37–2.13	33.3	2.2				
Hawaiian/Pacific Islander	5.01	3.35–7.49	57.1	4.5	7.15	5.03–10.15	77.8	2.6
Other APIs^2^	2.45	1.91–3.13	40.9	2.7	1.35	1.15–1.58	42.7	1.2
Duration of residence in the USA (years)								
<1	0.54	0.43–0.68	32.6	2.5	0.49	0.31–0.79	44.0	5.4
1–5	0.69	0.60–0.80	37.8	1.5	0.60	0.50–0.72	48.5	2.1
5–9	0.76	0.69–0.84	39.8	1.1	0.61	0.52–0.72	49.0	1.9
10–14	0.81	0.73–0.90	41.3	1.1	0.71	0.61–0.82	52.3	1.8
15+	0.90	0.85–0.95	43.5	0.6	0.81	0.75–0.88	55.5	0.9
US-born	1.00	Reference	45.9	0.2	1.00	Reference	60.1	0.2
Age (years)								
18–24	1.00	Reference	28.3	0.4	1.00	Reference	39.4	0.7
25–34	1.77	1.70–1.85	40.4	0.3	1.95	1.82–2.08	54.9	0.5
35–44	2.39	2.29–2.50	47.2	0.3	2.72	2.52–2.94	62.5	0.5
45–54	3.23	3.09–3.38	54.2	0.3	3.05	2.84–3.28	65.0	0.4
55–64	3.38	3.22–3.55	55.2	0.3	3.47	3.23–3.74	67.8	0.4
65+	2.36	2.25–2.48	47.0	0.3	2.45	2.27–2.64	60.2	0.5
Gender								
Male	1.00	Reference	57.1	0.2	1.00	Reference	68.3	0.3
Female	0.39	0.38–0.40	35.1	0.2	0.46	0.44–0.48	50.9	0.3
Education (years of school completed)								
0–8	1.55	1.48–1.62	50.3	0.5	1.40	1.26–1.54	61.2	1.0
9–11	1.49	1.43–1.56	49.4	0.4	1.35	1.26–1.44	60.4	0.7
12	1.36	1.32–1.39	47.2	0.2	1.49	1.43–1.56	62.7	0.4
13–15	1.26	1.22–1.29	45.5	0.3	1.46	1.40–1.52	62.1	0.4
16+	1.00	Reference	40.4	0.3	1.00	Reference	53.7	0.4
Poverty status (ratio of family income to poverty threshold)^3^								
<100%	1.26	1.15–1.38	48.4	1.0	1.11	1.03–1.19	60.2	0.7
100–199%	1.22	1.17–1.28	47.8	0.4	1.17	1.11–1.24	61.4	0.6
200–299%	1.21	1.17–1.26	47.6	0.3	1.14	1.08–1.21	60.9	0.5
300–399%	1.20	1.16–1.24	47.4	0.3	1.13	1.06–1.20	60.7	0.6
400–499%	1.16	1.13–1.19	46.5	0.3	1.09	1.03–1.15	59.9	0.5
≥500%	1.00	Reference	43.3	0.3	1.00	Reference	58.0	0.4

OR: odds ratio; SE: standard error; CI: confidence interval.

^1^Adjusted prevalence was derived from fitted logistic models that included survey year, age, gender, race/ethnicity, length of immigration, marital status, region of residence, education, and poverty status (or family income and family size).

^2^Other Asian categories include the Koreans, the Vietnamese, the Japanese, the Cambodians, the Laotians, the Hmongs, the Thais, the Pakistanis, and other Asian groups.

^3^The income categories during 1992–1995 were <7000, 7000–14999, 15000–24999, 25000–34999, 35000–49999, and 50000+. Family income, instead of poverty status, was used for the 1992–1995 NHIS.

**Table 4:** Observed and adjusted prevalence and odds of obesity and overweight and mean body mass index (BMI) among 12 ethnic-immigrant groups: United States, 2006–2011.

Ethnic-immigrant group	Observed prevalence^1^		Adjusted odds ratio^2^		Adjusted prevalence^2^		BMI	
	%	SE	OR	95% CI	%	SE	Mean	SE
	Obesity						Observed BMI	

Non-Hispanic white, US-born	26.4	0.2	9.49	6.48–13.89	26.1	0.2	27.4	0.0
Non-Hispanic white, immigrant	19.0	0.8	6.67	4.51–9.87	20.1	0.8	26.3	0.1
Chinese, US-born	8.3	1.6	3.48	1.97–6.15	11.7	2.1	24.4	0.2
Chinese, immigrant	3.3	0.6	1.00	Reference	3.7	0.7	23.1	0.2
Asian Indian, US-born	11.3	3.1	5.54	2.74–11.24	17.4	4.2	24.3	0.6
Asian Indian, immigrant	10.3	1.2	3.75	2.40–5.87	12.5	1.3	24.9	0.1
Filipino, US-born	22.3	1.9	8.79	5.66–13.65	24.8	1.9	26.7	0.3
Filipino, immigrants	13.5	1.3	4.61	3.00–7.08	14.9	1.3	25.6	0.2
Hawaiian/Pacific Islander, US-born	41.1	3.7	21.93	13.86–34.68	44.3	3.5	29.8	0.9
Pacific Islander, immigrant	36.8	6.4	14.23	7.38–27.46	34.4	6.3	30.1	1.6
Other Asians, US-born^3^	13.8	1.2	5.62	3.70–8.55	17.6	1.6	25.4	0.2
Other Asians, immigrant^3^	6.2	0.6	1.76	1.12–2.78	6.4	0.6	23.6	0.1

	Overweight						Adjusted BMI^2^	

Non-Hispanic white, US-born	61.1	0.2	4.47	3.78–5.29	60.8	0.2	27.3	0.0
Non-Hispanic white, immigrant	55.0	0.9	3.63	3.03–4.36	56.1	0.9	26.4	0.1
Chinese, US-born	38.1	2.8	2.43	1.82–3.24	46.9	2.8	25.5	0.2
Chinese, immigrant	25.2	1.5	1.00	Reference	27.8	1.6	23.4	0.2
Asian Indian, US-born	31.4	5.1	2.37	1.49–3.78	46.3	5.3	25.9	0.5
Asian Indian, immigrant	45.7	1.5	2.70	2.24–3.26	49.3	1.6	25.5	0.1
Filipino, US-born	57.6	2.4	4.66	3.66–5.93	61.7	2.4	27.2	0.3
Filipino, immigrant	51.0	1.8	3.20	2.59–3.94	53.2	1.6	25.9	0.2
Hawaiian/Pacific Islander, US-born	72.4	4.3	10.37	6.61–16.25	77.2	3.6	30.3	0.8
Pacific Islander, immigrant	76.3	4.8	9.10	4.95–16.73	75.0	5.1	29.8	1.6
Other Asians, US-born^3^	45.9	1.7	3.16	2.50–3.99	52.9	1.8	26.3	0.2
Other Asians, immigrant^3^	32.3	1.2	1.35	1.11–1.65	33.8	1.2	23.7	0.1

OR: odds ratio; SE: standard error; CI: confidence interval.

^1^Weighted prevalence.

^2^Adjusted prevalence was derived from fitted logistic or OLS regression models that included survey year, age, gender, ethnic-immigrant status, marital status, region of residence, education, and family income/poverty status.

^3^Other Asian categories include the Koreans, the Vietnamese, the Japanese, the Cambodians, the Laotians, the Hmongs, the Thais, the Pakistanis, and other Asian groups.

**Table 5:** Observed and adjusted prevalence and odds of obesity and overweight and mean body mass index (BMI) among 18 joint racial/ethnic and socioeconomic status (SES) groups: United States, 2006–2011.

Racial/ethnic-SES group	Observed prevalence^1^		Adjusted odds ratio^2^		Adjusted prevalence^2^		BMI	
	%	SE	OR	95% CI	%	SE	Mean	SE
	Obesity						Observed BMI	

Non-Hispanic white, low SES	32.5	1.1	8.15	4.32–15.36	32.9	1.2	28.2	0.2
Non-Hispanic white, middle SES	28.3	0.3	6.45	3.45–12.07	28.1	0.3	27.6	0.0
Non-Hispanic white, high SES	19.8	0.4	3.61	1.94–6.71	18.1	0.4	26.5	0.1
Chinese, low SES	4.7	2.6	1.47	0.38–5.71	8.4	4.6	23.1	0.4
Chinese, middle SES	5.0	0.9	1.46	0.69–3.09	8.3	1.4	23.6	0.2
Chinese, high SES	4.3	1.3	1.00	Reference	5.9	1.7	23.0	0.2
Asian Indian, low SES	7.9	6.6	2.06	0.30–13.99	11.3	9.1	25.8	0.8
Asian Indian, middle SES	14.7	2.1	5.55	2.80–10.99	25.2	3.2	25.0	0.2
Asian Indian, high SES	7.0	1.1	2.13	1.04–4.38	11.7	1.9	24.5	0.2
Filipino, low SES	29.2	7.0	12.38	4.87–31.43	42.3	8.0	27.7	0.9
Filipino, middle SES	18.9	1.6	5.63	2.91–10.90	25.5	1.9	26.2	0.2
Filipino, high SES	13.0	2.0	3.10	1.50–6.43	16.0	2.4	25.8	0.3
Hawaiian/Pacific Islander, low SES	20.1	11.2	8.75	1.80–42.58	34.4	16.1	27.2	1.4
Hawaiian/Pacific Islander, middle SES	38.5	4.0	14.69	7.03–30.70	46.4	4.2	30.2	1.0
Hawaiian/Pacific Islander, high SES	17.2	8.3	4.17	1.20–14.52	20.3	9.1	26.1	1.0
Other Asians, low SES^3^	9.0	2.3	2.82	1.24–6.42	14.8	3.4	24.8	0.4
Other Asians, middle SES^3^	8.5	0.7	2.37	1.27–4.40	12.7	1.1	24.2	0.1
Other Asians, high SES^3^	6.1	1.1	1.38	0.63–3.00	7.9	1.4	23.7	0.2

	Overweight						Adjusted BMI^2^	

Non-Hispanic white, low SES	61.3	1.2	4.86	3.65–6.47	63.1	1.3	28.3	0.2
Non-Hispanic white, middle SES	62.5	0.3	4.78	3.68–6.20	62.7	0.3	27.6	0.0
Non-Hispanic white, high SES	56.8	0.5	3.10	2.38–4.03	52.9	0.5	26.0	0.1
Chinese, low SES	27.5	5.4	1.46	0.79–2.68	35.8	6.1	24.6	0.4
Chinese, middle SES	31.0	1.9	1.79	1.33–2.42	40.4	2.0	25.1	0.2
Chinese, high SES	24.4	2.3	1.00	Reference	28.2	2.5	23.7	0.2
Asian Indian, low SES	62.9	9.5	5.73	2.37–13.82	66.5	9.0	26.6	0.8
Asian Indian, middle SES	44.8	2.4	3.67	2.71–4.97	56.8	2.3	26.7	0.2
Asian Indian, high SES	40.7	2.2	2.45	1.74–3.46	47.5	2.5	25.7	0.2
Filipino, low SES	63.6	7.5	8.59	4.00–18.45	74.4	6.4	29.3	0.9
Filipino, middle SES	54.4	1.7	4.49	3.35–6.01	61.3	1.6	27.2	0.2
Filipino, high SES	51.4	2.7	3.49	2.49–4.89	55.7	2.5	26.4	0.3
Hawaiian/Pacific Islander, low SES	73.6	13.3	13.30	4.17–42.39	81.4	8.2	29.0	1.3
Hawaiian/Pacific Islander, middle SES	72.5	3.4	11.08	6.99–17.57	78.7	2.9	31.2	1.0
Hawaiian/Pacific Islander, high SES	57.0	10.9	4.79	2.05–11.16	62.7	9.2	26.7	0.8
Other Asians, low SES	44.1	5.0	3.12	1.84–5.30	53.1	5.2	26.1	0.4
Other Asians, middle SES	35.8	1.3	2.12	1.59–2.82	44.2	1.4	25.4	0.1
Other Asians, high SES	33.1	2.4	1.49	1.07–2.07	36.3	2.4	24.2	0.2

OR: odds ratio; SE: standard error; CI: confidence interval. Low SES: education <12 years and family income below poverty level. High SES: education ≥16 years and family income ≥400% of poverty threshold.

^1^Weighted prevalence.

^2^Adjusted prevalence was derived from fitted logistic or OLS regression models that included survey year, age, gender, racial/ethnic-socioeconomic group, marital status, and region of residence.
